# Supplementary material for: Flipping the curriculum for resident didactics: In-Training and certifying examination scores in an internal medicine residency
Source: PLoS One. 2025 Jul 8;20(7):e0327655. doi: 10.1371/journal.pone.0327655 (PMC12237032; doi:10.1371/journal.pone.0327655)
Supplement: Attachment — Submitted filename: Flipping the Curriculum Response to Reviewers.docx [file pone.0327655.s002.docx]

***Reviewer Comments to Author:***

***Reviewer #1: Thank you for submitting this very interesting article entitled Flipping the Curriculum for Resident Didactics: In-Training and Certifying Examination Scores in Internal Medicine Residency. This article hypothesizes that widespread implementation of a flipped curriculum approach in resident didactics would result in improved knowledge acquisition and retention as reflected on standardized examination scores.***

***Overall the article is good but requires some major revisions. These following suggestions may help the authors:***

***Abstract:***

***1. The first sentence remove the word “that”. There are some grammatical/english sentence structure issues. Suggest careful proofreading.***

“That” was removed from the first sentence and the manuscript peer-reviewed for grammatical issues.

***Introduction:***

***1. please add more information and a better description of “flipped learning”. While traditional classroom education is well-known, most readers would benefit from some additional clarification. Since the period covers the COVID pandemic, some information regarding how the program dealt with this (and how the data was calculated/interpreted) is necessary to address (perhaps in methods and discussion).***

More information was added to the introduction on flipped learning.

Citation: Gilboy MB, Heinerichs S, Pazzaglia G. Enhancing student engagement using the flipped classroom. *J Nutr Educ Behav*. 2015;47(1):109-114. doi:10.1016/j.jneb.2014.08.008

The COVID pandemic has been clarified and reviewed in the discussion:

“Fifth, the COVID-19 pandemic affected program implementation and may have impacted test scores.^41^ ^41^ All in-person conferences and learning activities were suspended from March 16, 2020 to July 1, 2020, starting with the COVID shutdown in Ohio. Also, IM-ITE and IM-CE performance may have suffered from interrupted organized group sessions, potentially hiding what would have otherwise been an even stronger positive signal from the curricular change.”

***2. Third paragraph, please cite where the recent systematic review identifying only 22 articles took place (PubMed?)***

King AM, Gottlieb M, Mitzman J, Dulani T, Schulte SJ, Way DP. Flipping the Classroom in Graduate Medical Education: A Systematic Review. *J Grad Med Educ*. 2019;11(1):18-29. doi:10.4300/JGME-D-18-00350.2

This was a review of multiple databases including pubmed, CINAHL Plus, Embase, Web of Science Core Collection, and ERIC.

This has been clarified in the text.

***Methods:***

***1. One weakness of the study involves comparing a cohort of three years (2014-2017) to those after the redesign of the curriculum. Are these groups comparable? The figure is very helpful in understanding the distribution, but does not render well in black and white.***

We ran an independent samples t-test and chi-squared test. Rather than change at the individual level, we assessed for program-level change. We endeavored to pragmatically assess the influence of our flipped classroom implementation.

***2. it seems that in the flipped classroom, residents come much more prepared to discuss the content then in the traditional classroom. This may confound the results found in the study. It would be interesting to know if any pretest was conducted before sessions.***

That is an interesting point. We collected data for the performance on the validated IM-ITE and ABIM for 3 years pre-, during, and 3 years post- implementation. We did not have a in-house validated pre-assessment. We endeavored to test whether this flipped classroom educational methodology contributed to improved knowledge acquisition. It is probably worth mentioning as well that better pre-class preparation is one of the components of the FC approach.

***3. there is mention of a 50-minute primer presentation. Is this in the format lecture? In other words, are learners now receiving both a lecture, as well as an interactive time for discussion?***

There is a 15-minute primer presentation that reviews the same learning objectives as the PBL session, in lecture format. This was our method to ensure that the audience had the scaffolding necessary to effectively engage in flipped classroom learning.

The primer is discussed in our methods section:

“Three residents collaboratively prepare a 15-minute primer presentation that contains foundational knowledge for the noon conference of the day.”

We added lectures to our introduction, as well:

“Pre-class learning takes many diverse forms, including lectures, video lectures, podcasts, vodcasts, textbook readings, and journal articles.”

And explored this in our discussion:

“Given their patient care responsibilities, residents have limited time and energy to acquire foundational knowledge outside the classroom. Our residents were instructed to read preparatory articles and textbook chapters, and many reported being ready for flipped conference activities and each block’s TBL. This may represent the impact of re-imagined morning report sessions, which we call the Resident-led Educational Primer. This adaptation was particularly well-received by residents, who voiced that they felt more prepared and able to work collaboratively in the PBL sessions. Furthermore, to our knowledge, this innovation has not been described before in literature. It uniquely and elegantly addresses a principal concern about FC’s feasibility in GME: the difficulty in guaranteeing the crucial pre-class component for busy residents.”

***4. Are the educator team (chief residents, attendings, etc..) consistent for each session and are they the same educators from the years before the change in curriculum?***

The educator team remained similar. While chiefs change over each academic year, program leadership and core faculty were consistent.

***5. The statistical analysis is adequate but detail showing that the groups are comparable/racial/gender differences is suggested***

This is an area of future research, that we will explore in our discussion. This data did not align with our research question or hypothesis, when we wrote our research proposal. Our intent way to pragmatically evaluate the impact of a wholesale transition to flipped learning.

“Third, we did not examine the effect of demographic characteristics (e.g., sex, race, ethnicity) or standardized testing information before residency. It has been postulated that flipped classrooms and active learning may have extra benefits for underrepresented students, who may perform better in more interpersonal environments with more faculty-learner interaction”

***Discussion***

***1. There is mention of “longer term knowledge retention” but the data does not show this.***

Great point, this was conjecture, we removed the “longer term” qualifier.

***2. How many residents actually prepared for the session is unclear and this may affect results. It is hard to believe that all residents, who are so busy, would have time to consistently prepare. Also the effects of self-preparing and reading, the 50-minute primer presentation and the reverse classroom discussion build and reinforce each other. This point may need to be made more specifically.***

Thank you for the feedback. We did not measure resident preparation. The 15-minute primer presentation was built into daily schedules, provides necessary scaffolding, and is a unique approach to guaranteeing the pre-classroom component in a GME environment.

“This adaptation was particularly well-received by residents, who voiced that they felt more prepared and able to work collaboratively in the PBL sessions. Furthermore, to our knowledge, this innovation has not been described before in literature. It uniquely and elegantly addresses a principal concern about FC’s feasibility in GME: the difficulty in guaranteeing the crucial pre-class component for busy residents.”

***3. The two groups contain an unequal number of residents, and the demographics of the residents may not be the same. The question as to if the 2 groups are comparable should be further discussed in the limitations.***

We studied our entire population of residents that trained in our program from 2014-2021. We did not design the study to assess for demographics, but given our somewhat homogenous population, I doubt we would be powered for significance. We did not complete an a-priori power analysis on demographic characteristics, as our research question was population-level.

***4. The data shows no difference in im-ce, this should be furthered addressed in the discussion.***

Thank you. I have added this to the discussion.
