# Supplementary material for: Flipping the curriculum for resident didactics: In-Training and certifying examination scores in an internal medicine residency
Source: PLoS One. 2025 Jul 8;20(7):e0327655. doi: 10.1371/journal.pone.0327655 (PMC12237032; doi:10.1371/journal.pone.0327655)
Supplement: Attachment — Submitted filename: Response to reviewers _10_4_2024.docx [file pone.0327655.s003.docx]

Review Comments to the Author

Please use the space provided to explain your answers to the questions above. You may also include additional comments for the author, including concerns about dual publication, research ethics, or publication ethics. (Please upload your review as an attachment if it exceeds 20,000 characters)

Reviewer #1: Thank you for submitting this revised manuscript. This draft reads much better and most of our suggestions were well integrated into the new manuscript. While this draft is suitable for publication, we have a few minor suggestions.


Introduction:
1. 1st sentence based on lectures” -> Lecture based.

**addressed**
2. “with peers and instructors to apply the same concepts”  the concept or these concepts

**Thank you. These sentences have been reworked to address the points raised by reviewer 3.**
3. “GME has been rather limited”, consider adding “because….(reasons)”
**Certainly, this is intriguing. I have some thoughts but would not feel comfortable speculating here.**
Results:
Consider moving the explanation of effect size and cohen’s d to the discussion.
**This section has been moved to the discussion.**

Discussion:
“professed claims” (consider removing professed).
**This has been removed as suggested**
Consider adding what the future direction of the studies might be to help answer some of the questions that this study raises. It may be interesting to know how long residents spent preparing for the FC at home as well as the consistency of being able to prepare given the time commitments of residency.
**A paragraph addressing these points has been added to the discussion**

Conclusion:
“before and after”.
**This has been changed as suggested**
Figure 1: The black and white table does nor render well. Consider changing “X” for LB, Transition, FC.
**These have been changed as suggested**
Table 1: Can we add range, SEM to the means and N?

**We temporarily accessed the data and do not presently have access to the data.**

Reviewer #2: Thank you for submitting this very valuable paper. This is a very valuable paper that demonstrates the usefulness of Flipping the Curriculum in resident education. I consider that the authors have responded sincerely and adequately to the points raised by the reiewer.

Reviewer #3: Abstract: Succinctly written and reflects the content of the manuscript.

Introduction:
Colloquialisms such as “lay down” in the sentence “Learners must spend time and energy to lay down conceptual frameworks…” should be avoided and replaced with more precise language. Also, with regards to that sentence, I do not understand the authors use of the phrase “conceptual frameworks” in this context. The sentence that follows (“This frees the cognitive capacity classroom learners…”) also needs to be modified as it does not make sense as it is currently written.

The authors have neglected to mention a major advantage of the flipped classroom, which is that, ideally, all learners walk into the classroom with the same baseline level of knowledge. More junior learners will likely need to spend more time with the pre-classroom work than more senior learners, but they should all walk in with the same level of knowledge once that pre-work is completed.
**Thank you for these points. These sentences have been reworked to avoid colloquialism, improve clarity, and to address this additional major advantage of the flipped classroom.**

Methods:
The methods of the study are explained clearly.

Do the authors monitor whether the residents are completing the reading and MKSAP questions prior to morning report?
**This is now addressed in the discussion by informing the reader that reports of resident preparedness were anecdotal.**
I do wonder what the impetus was for changing to the FC method in the first place. Was it because IM-ITE and IM-CE scores were poor or in need of improvement? If that is the reason for changing to a FC format, then those would be reasonable outcomes to look at. If, however, the change to FC was made for some other reason like low attendance or low satisfaction with the didactics, then those would be better outcomes to measure. It would be helpful if the authors clarified WHY they changed to a FC model, preferably in the introduction, so they can justify their use of exam scores as an outcome.

**Excellent point, the last paragraph of the introduction has been altered to reflect our motivations for looking at knowledge acquisition outcomes.**


Results:
The authors should refrain from analyzing the data or making editorial comments (for example, “…educational researchers know that effect size is more informative than statistical significance” or explanation of the definition and importance of effect size) and just report the data in the results section. Commentary should be provided in the discussion section, not here.
**This section has been moved to the discussion.**
Otherwise, the results are presented clearly.


Discussion:
The first paragraph is all over the place and needs to be written more clearly. For example, the authors write “They [residents] must routinely practice on-the-spot critical thinking. Others have shown that…”; who are the “Others” the authors refer to? These sentences don’t seem to follow together naturally.

**The first paragraph has been rewritten following the suggestion above to move the commentary on effect size to the discussion.**
The authors state “Our residents were instructed to read preparatory articles and textbook chapters, and many reported being ready for flipped conference activities and each block’s TBL.” How do the authors know this? Did they collect data about how prepared the residents felt, or is this anecdotal? If anecdotal, the authors need to make this clear. If there are data to support this statement, the authors need to provide these data.
**This has been changed to reflect that this assertion is drawn from anecdotes**
The authors introduce the novelty of the reimagined morning report sessions, which is great, but seems like it should be an entirely different paper and not addressed here. It detracts from the actual research conducted and presented in this manuscript.
**Thank you for this input. The discussion of these sessions has been scaled back substantially**
In the final paragraph, I do not know what the authors mean by “…performance may have suffered from interrupted organized group sessions…”.
**This has been clarified**
A major limitation not mentioned by the authors is that you really cannot firmly apply cause and effect to the FC and change in examination scores. There are many, many other variables that could impact examination scores. Were new faculty hired who were excellent teachers? Were the newer residents somehow more inherently motivated to read and learn than the previous residents? Who knows. The FC certainly may have contributed to increased scores, but the cause and effect is certainly not 100%.
**This point is well received and has been included in the discussion of limitations.**

Citations: I do not see a #7 in the list of citations, this should be added.

**#7 has been added to the corresponding article.**

Reviewer #4: The responses to the prior reviewer have been addressed.
There are a few typographical errors to be addressed.
1. Under introduction. The header "Introduction" should be moved before the start of the first paragraph.

**It has been moved as suggested**
2. In the paragraph beginning with "Residency didactic education" in the introduction the 9th sentence "This frees the cognitive capacity" replace the next word "classroom" with "for".

**This has been changed as suggested**
3. In methods 5th sentence, 1st paragraph insert a comma after "workgroup" and one after "faculty".

**This has been changed as suggested**
4. Need space between "typically" and "meet" in the 8th sentence of 1st paragraph

**This has been changed as suggested**
