# Supplementary material for: Flipping the curriculum for resident didactics: In-Training and certifying examination scores in an internal medicine residency
Source: PLoS One. 2025 Jul 8;20(7):e0327655. doi: 10.1371/journal.pone.0327655 (PMC12237032; doi:10.1371/journal.pone.0327655)
Supplement: Attachment — Submitted filename: Flipping the Curriculum comments for authors _6_12_2025.docx [file pone.0327655.s005.docx]

Additional Editor Comments:

Before I can accept this for publication I request one minor revision: please carefully revise the abstract to ensure the findings are clearly presented. There are grammatical errors in places and some ambiguity in how the findings in particular are presented. It may be helpful to ask a trusted colleague to support in revising the abstract to enhance the clarity overall. I make this request as the abstract gives readers the first impression of an article and they are more likely to read and use your work if the abstract flows well and clearly gives all the necessary information.
**The abstract has been rewritten to improve the grammar, clarity, and readability.**

[Note: HTML markup is below. Please do not edit.]

Reviewers' comments:

Reviewer's Responses to Questions

**Comments to the Author**

1. If the authors have adequately addressed your comments raised in a previous round of review and you feel that this manuscript is now acceptable for publication, you may indicate that here to bypass the “Comments to the Author” section, enter your conflict of interest statement in the “Confidential to Editor” section, and submit your "Accept" recommendation.

Reviewer #1: All comments have been addressed

Reviewer #4: All comments have been addressed

2. Is the manuscript technically sound, and do the data support the conclusions?

The manuscript must describe a technically sound piece of scientific research with data that supports the conclusions. Experiments must have been conducted rigorously, with appropriate controls, replication, and sample sizes. The conclusions must be drawn appropriately based on the data presented.

Reviewer #1: Yes

Reviewer #4: Yes

3. Has the statistical analysis been performed appropriately and rigorously?

Reviewer #1: Yes

Reviewer #4: Yes

4. Have the authors made all data underlying the findings in their manuscript fully available?

The [PLOS Data policy](https://nam04.safelinks.protection.outlook.com/?url=http%3A%2F%2Ftrack.editorialmanager.com%2FCL0%2Fhttp%3A%252F%252Fwww.plosone.org%252Fstatic%252Fpolicies.action%2523sharing%2F1%2F010f0196d1adec6b-1cc69189-9faf-46cc-9ca8-7aaec032bb97-000000%2Flx5gjC6sRF5nzLNxcxLA327kB1rllEfy0LTeTNPmZAc%3D211&data=05%7C02%7Cluke.mccoy%40wright.edu%7C78013a267311424b602a08dd9354921f%7C5c46d65dee5c45138cd4af98d15e6833%7C0%7C0%7C638828713655263622%7CUnknown%7CTWFpbGZsb3d8eyJFbXB0eU1hcGkiOnRydWUsIlYiOiIwLjAuMDAwMCIsIlAiOiJXaW4zMiIsIkFOIjoiTWFpbCIsIldUIjoyfQ%3D%3D%7C0%7C%7C%7C&sdata=vlwDQbMpaczC6iQdgP4iJWBFf0wxUN9WOB8RnJLgAgo%3D&reserved=0) requires authors to make all data underlying the findings described in their manuscript fully available without restriction, with rare exception (please refer to the Data Availability Statement in the manuscript PDF file). The data should be provided as part of the manuscript or its supporting information, or deposited to a public repository. For example, in addition to summary statistics, the data points behind means, medians and variance measures should be available. If there are restrictions on publicly sharing data—e.g. participant privacy or use of data from a third party—those must be specified.

Reviewer #1: Yes

Reviewer #4: Yes

5. Is the manuscript presented in an intelligible fashion and written in standard English?

PLOS ONE does not copyedit accepted manuscripts, so the language in submitted articles must be clear, correct, and unambiguous. Any typographical or grammatical errors should be corrected at revision, so please note any specific errors here.

Reviewer #1: Yes

Reviewer #4: Yes

6. Review Comments to the Author

Please use the space provided to explain your answers to the questions above. You may also include additional comments for the author, including concerns about dual publication, research ethics, or publication ethics. (Please upload your review as an attachment if it exceeds 20,000 characters)

Reviewer #1: The authors have done an excellent job at implementing all suggested changes and addressing all reviewer concerns. This shortened version reads well. I believe that this version is ready for publication as is. Some things to consider is to add SEMs on table 1 to show the tightness of the distribution and to remove the colors on page 6 in materials and methods (figure 1 is self explanatory regarding color). Well done.

Reviewer #4: (No Response)

**The colors discussion on page 6 in materials and methods has been removed.**

7. PLOS authors have the option to publish the peer review history of their article ([what does this mean?](https://nam04.safelinks.protection.outlook.com/?url=https%3A%2F%2Ftrack.editorialmanager.com%2FCL0%2Fhttps%3A%252F%252Fjournals.plos.org%252Fplosone%252Fs%252Feditorial-and-peer-review-process%2523loc-peer-review-history%2F1%2F010f0196d1adec6b-1cc69189-9faf-46cc-9ca8-7aaec032bb97-000000%2F50RyyPUVnCWV9irAp2vhKbhkjN0I8ujSjBJiFQL6bfA%3D211&data=05%7C02%7Cluke.mccoy%40wright.edu%7C78013a267311424b602a08dd9354921f%7C5c46d65dee5c45138cd4af98d15e6833%7C0%7C0%7C638828713655298635%7CUnknown%7CTWFpbGZsb3d8eyJFbXB0eU1hcGkiOnRydWUsIlYiOiIwLjAuMDAwMCIsIlAiOiJXaW4zMiIsIkFOIjoiTWFpbCIsIldUIjoyfQ%3D%3D%7C0%7C%7C%7C&sdata=aK%2Bf5hog%2Bps7xXVXwFPLhb88PQx0o3WJ7KzqapEz6SE%3D&reserved=0)). If published, this will include your full peer review and any attached files.


If you choose “no”, your identity will remain anonymous but your review may still be made public.


**Do you want your identity to be public for this peer review?** For information about this choice, including consent withdrawal, please see our [Privacy Policy](https://nam04.safelinks.protection.outlook.com/?url=https%3A%2F%2Ftrack.editorialmanager.com%2FCL0%2Fhttps%3A%252F%252Fwww.plos.org%252Fprivacy-policy%2F1%2F010f0196d1adec6b-1cc69189-9faf-46cc-9ca8-7aaec032bb97-000000%2FnPChGFzFyV6F0C7ChJgGBRyKAUQeFseAq6QKKyqD7Uw%3D211&data=05%7C02%7Cluke.mccoy%40wright.edu%7C78013a267311424b602a08dd9354921f%7C5c46d65dee5c45138cd4af98d15e6833%7C0%7C0%7C638828713655327517%7CUnknown%7CTWFpbGZsb3d8eyJFbXB0eU1hcGkiOnRydWUsIlYiOiIwLjAuMDAwMCIsIlAiOiJXaW4zMiIsIkFOIjoiTWFpbCIsIldUIjoyfQ%3D%3D%7C0%7C%7C%7C&sdata=rQoI65LUP7q0c7rFHSKzimBazZS7QJ1mcjeGQYh2LiU%3D&reserved=0).

Reviewer #1: No

Reviewer #4: No
